# Seismic rate variations prior to the 2010 Maule, Chile M_W_ 8.8 giant megathrust earthquake

**DOI:** 10.1038/s41598-021-82152-0

**Published:** 2021-02-01

**Authors:** Benoit Derode, Raúl Madariaga, Jaime Campos

**Affiliations:** 1 Department of Geophysics (DGF), University of Chile, Blanco Encalada 2002, Santiago, Chile; 2 Laboratoire de Géologie, PSL University, Ecole Normale Supérieure et CNRS, 75230 Paris, France

**Keywords:** Seismology, Tectonics

## Abstract

The M_W_ 8.8 Maule earthquake is the largest well-recorded megathrust earthquake reported in South America. It is known to have had very few foreshocks due to its locking degree, and a strong aftershock activity. We analyze seismic activity in the area of the 27 February 2010, M_W_ 8.8 Maule earthquake at different time scales from 2000 to 2019. We differentiate the seismicity located inside the coseismic rupture zone of the main shock from that located in the areas surrounding the rupture zone. Using an original spatial and temporal method of seismic comparison, we find that after a period of seismic activity, the rupture zone at the plate interface experienced a long-term seismic quiescence before the main shock. Furthermore, a few days before the main shock, a set of seismic bursts of foreshocks located within the highest coseismic displacement area is observed. We show that after the main shock, the seismic rate decelerates during a period of 3 years, until reaching its initial interseismic value. We conclude that this megathrust earthquake is the consequence of various preparation stages increasing the locking degree at the plate interface and following an irregular pattern of seismic activity at large and short time scales.

## Introduction

Giant subduction earthquakes are the result of a long-term stress localization due to the relative movement of two adjacent plates. Before a large earthquake, the interface between plates is locked and concentrates the external forces, until the rock strength becomes insufficient, initiating the sudden rupture along the plate interface. Giant Megathrust Earthquakes (GME) correspond to the rapid stress release and slip at the shallow part of the plate interface. Whereas large earthquakes can occur between two strike-slip plates, such as the great San Francisco earthquake of 1906 (USA, M_W_ 7.9) or the Izmit 1999 earthquake (Turkey, M_W_ 7.7)^1^, the giant earthquakes (M_W_ > 8.5, 16 world occurrences since 1900) involve large scale rupture of the interface between two convergent plates, with one plate subducting beneath another.

The great 2010 Maule earthquake took place in a region that had been studied over a period of 15 years before the main event by seismic and geodetic methods^2–6^. These authors demonstrated that during the period preceding the main event, the two plates were strongly coupled and that very few events occurred in the area of the main event before 2009. In a preliminary assessment of the Maule event^2^, it was reported that the main event was preceded by a small number of foreshocks during the 3 months preceding the main event (four M > 4.5). The Maule earthquake had also a strong aftershock activity that has been studied by numerous authors^7–11^.

The preparation phase of strain accumulation at the interface, and its subsequent major release, is known as the seismic cycle and is macroscopically controlled by the strain accumulation rate and the strength to failure at the interface^12–14^. In practice, a large number of parameters can influence the regularity of the cycle, as a number of space and time scales are involved and the first approximation of elastic surrounding materials (crusts, plates) is somewhat simplistic. Only a few examples of observed seismic cycles are documented^15,16^, where the interseismic period corresponds to low seismic activity or aseismic faulting followed (or not) by brittle seismic ruptures preceding the main event. These foreshocks, corresponding to bursts of seismic activity occurring in a short time before a large earthquake and close to its rupture zone, have been frequently observed in various zones and contexts (intraplate/interplate^17–19^, subduction earthquakes^3,20^), but their mechanical connection with the large earthquakes that follow are still unclear^21–23^. Bouchon et al*.*^24^ proposed, using observations of the 2011 M_W_ 9 Tohoku and the 2014 M_W_ 8.2 Iquique earthquakes, that foreshock seismic activity down-dip in the slab below the seismogenic zone could reflect a tensional deformation at depth prior to major interplate earthquakes leading to the nucleation of the initiation phase of the main earthquake dramatic rupture. Recent observations tend to show that in some cases, a short time before the main shock (from minutes to months before the main shock), slow slip events (SSE)^25–29^ within the seismogenic zone, or episodic tremors and slip (ETS)^30,31^ below the seismogenic zone may occur, indicating that unusual semi-aseismic slip can act as precursors of large ruptures. However, the irregular nature^3,31,32^ of the sequences of seismic activity followed or synchronized with aseismic slow slip at depth prohibits us from using them as an unambiguous proxy to predict brutal and large ruptures.

At larger time scales before the main seismic event, some author found that seismic rate changes can be observed in the whole coseismic area or in the vicinity of the rupture zone. Huang^33^ indicates for example that for some major earthquakes of the Asia and Asia–Pacific regions, a seismic quiescence, corresponding to a substantial seismic rate decreasing, occurs a few years before the mainshock. In the case of moderate earthquakes (5 < M_W_ < 6), Gentili et al*.*^34^ also found in most of their studied cases seismic quiescence processes, alternating with regain of seismic activity from months to a few years before the main event. As for the foreshock activity in a moderate to short time before the main event, the rapid quiescence of usually seismogenic zones could play the role of potential precursors of major ruptures. Various studies indicate the occurrence of seismic foreshocks (classic earthquakes, SSE, ETS), or possible shutdown of seismic activity for decades^35^ before large earthquakes, but still no observations of these combined seismic rate changes in the short time before a subduction Giant Mega-Earthquake (GME in the following) exist.

In the work presented here, we analyze seismic activity in the area of the 27 February 2010, M_W_ 8.8 Maule GME at different time scales from 2000 to 2019 in order to more precisely characterize the potential changes in the seismic rate preceding this earthquake. We differentiate the seismicity located inside the coseismic rupture zone of the main shock from that located in the areas surrounding the rupture zone, using equivalent spatial areas and similar time windows of observations. Our original combined method of spatial–temporal analysis of the seismicity within and around the coseismic rupture zone allows us to identify potential seismic precursors of the Maule earthquake, beginning years before the mainshock as well as a few days before it.

## Methods

### Geometry of the studied zone

**Figure 1:**
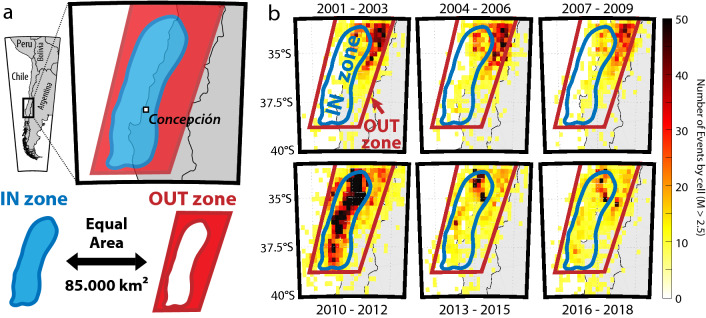
Studied area and associated seismic rate from 2000 to 2018. (**a**) Geometry of the studied zone. The Maule coseismic rupture area (IN-zone, blue patch, from Moreno et al*.*^36^) and the surrounding zone (OUT-zone, red patch) are area-equivalent and together contain 95% of the entire recorded seismicity within the region defined in (**b**) for the considered period. (**b**) Seismic density maps by sets of 3 years segments from 2001 to 2018. All the events of the catalog used are considered.

To analyze the seismic activity in the near area of the M_W_ 8.8 Maule earthquake, we differentiated two zones located within the 33°S–40°S/69°W–76°W region, corresponding to the center-coast of Chile (Fig. 1). The first zone (IN-zone, blue in the figure) was defined as the limit of the coseismic slip of the Maule Earthquake, obtained by Moreno et al*.*^36^ using joint inversion of GPS, InSAR and land-level changes^36,37^. This model was selected amongst the existing coseismic models for its robustness, using all the relevant GPS data available at this time. In order to study the specificity of the seismic activity of the Maule coseismic zone before and after the main shock, we defined an OUT-zone encompassing the seismicity outside the coseismic area, but inside the surrounding region (Fig. 1a). The OUT-zone had been chosen to have an equivalent area to the IN-zone for consistency of the comparisons. The geometry of the closed polygon delimitating the OUT-zone includes more than 95% of the seismicity in the region during the studied period.

### Earthquake catalogue

**Figure 2:**
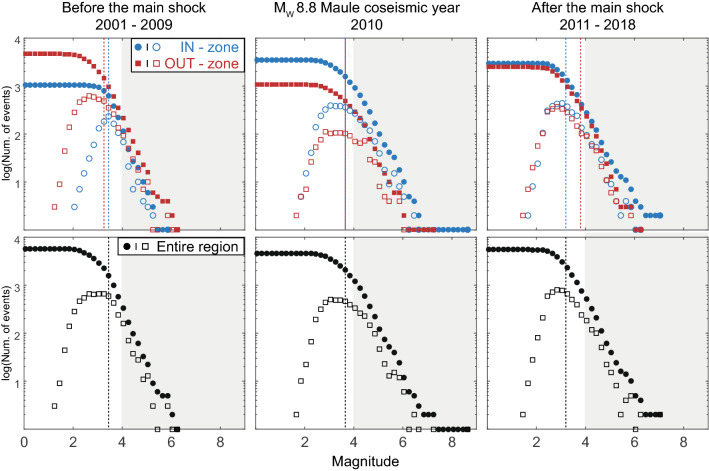
FMD variations of the ISC catalogue for the studied areas from 2000 to 2018. Cumulative FMD for the IN-zone dataset (filled blue circles), OUT-zone (filled red squares), and the entire region defined in Fig. 1 (filled black circles). The non-cumulative FMD are represented with the same color-code but with empty symbols (blue circles, red squares and black squares for the IN, OUT and entire region, respectively). Vertical dashed lines indicate the highest value of the magnitude of completeness *M*_*C*_ computed for each dataset using MAXC and MBS methods (see Methods section). The grey zones represent the magnitude range of the data used in this study (M > 4), which is larger than the local magnitude of completeness of the catalog.

We used the recent updated catalogue made by the International Seismological Center (ISC, http://www.isc.ac.uk, last reviewed on February 2017) to determine the location and magnitude of the events during the period 2000–2018 of the study. In order to maximize the coherence of the data during this period, we prioritized, when available, M_W_ and M_S_ magnitudes and location estimations of the ISC, GFZ, NEIC, GUC, and SJA respectively (see http://www.isc.ac.uk/iscbulletin/agencies/ for more information about the contributing agencies). In order to diminish the impact of the non-thrust seismicity on our observations (such as intra-accretionary wedge seismicity or intraplate seismicity), we only considered seismicity located at depths larger than 20 km. Figure 2 shows the cumulative and non-cumulative Frequency-Magnitude Density (FMD) of our seismic dataset for the three regions defined in Fig. 1b.

We evaluated the magnitude of completeness *M*_*C*_ of our datasets by using two specific catalog-based techniques, and selected for each dataset the highest value in order to comfort the selected range of magnitude used in this study: (1) the non-parametric maximum curvature method (MAXC), consisting in computing the maximum value of the first derivative of the FMD curve, and (2) the parametric magnitude of completeness estimation by b-value stability (MBS) defined by Cao and Gao^38^ and improved by Woessner et al*.*^39^. The MAXC technique is known to underestimate *M*_*C*_ in bulk data, whereas the MBS overestimates *M*_*C*_, so that computing both methods can provide a reliable range of *M*_*C*_ estimations^40^. The *b*-value is then computed by using the maximum likelihood method parametrized with the estimated *M*_*C*_:

1 $$b = \frac{{log_{10} \left( e \right)}}{{\left\langle M \right\rangle - \left( {M_{C} - \frac{\Delta M}{2}} \right)}}$$

where $\left\langle M \right\rangle$ corresponds to the mean magnitude of the sample for magnitudes higher than $M_{C}$, and $\Delta M$ to the binning width of the catalog^41^. The values of *M*_*C*_ obtained for each period and dataset with both MAXC and MBS methods and the corresponding *b*-values are shown in the supplementary material (Supplementary Table T1).

### Statistical analysis of the earthquakes sequence

In order to obtain a quantitative value reflecting if the seismicity observed within the IN-zone a few days before the 2010 main shock is connected or independent of the following major event, we compute a simple statistical test based on the random Poisson distribution. Basically, we calculate for our dataset the parameter λ of the Poisson distribution for different periods of time before the main event, and deduced the probability that the observed seismicity before the main shock occurs. If the probability is high, then the observed foreshock sequence is not specific to the following main shock, as such sequence could frequently and randomly occur. If it is low, then this type of sequence is rare or unique and could be related to the subsequent mainshock. The Poisson probability can be written as:

2 $$P\left( {N = k ;\lambda_{{{\Delta }t}} } \right) = \frac{{\lambda_{{{\Delta }t}}^{k} }}{k!}e^{{ - \lambda_{{{\Delta }t}} }}$$

where *N* is the number of events, $\lambda_{\Delta t} = S_{R} \times \Delta t$ with *S*_*R*_ the seismic rate for the chosen period of the dataset and *Δt* the period in which we evaluate the probability that *N* events occur. The probability that a sequence of at least *k* events occur within a period *Δt* for a selected dataset can then be written as:

3 $$P\left( t \right) = P\left( {N \ge k ;\lambda_{{{\Delta }t}} } \right) = 1 - \mathop \sum \limits_{i = 0}^{i = k - 1} P\left( {N = i ;\lambda_{{{\Delta }t}} } \right)$$

**Figure 3:**
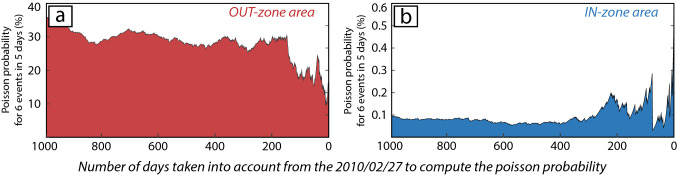
Poisson probability based on the frequency of earthquake occurrence. Probability that six earthquakes occurred in a time period of 5 days before the Maule main shock (see Fig. 5a), considering a time distribution of the events following a Poisson’s law. (**a**) Results using the OUT-zone region dataset, ranging from 20 to 35% that such a case happen. (**b**) Results using only events distributed within the IN-zone. In this case, there is only 0.1–0.3% that six earthquakes occur in 5 days within the IN-zone for all the time period tested.

Figure 3 shows the results of the Poisson probability *P*(*t*), function of the considered periods *t*, for *k* = 6 events occurring in *Δt* = 5 days for both studied regions. These results are discussed in the later part (see results and Fig. 5).

## Results

### Seismic quiescence

**Figure 4:**
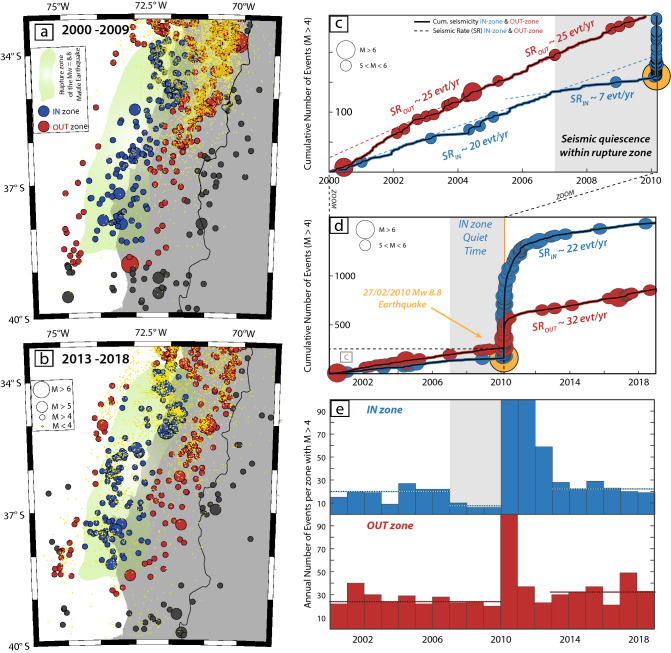
Seismic rate evolution of the 27F region before and after the main shock. Seismic activity recorded before (**a**) and after (**b**) the 27F event. The color code indicates the seismicity occurring within the coseismic zone (IN-zone, blue) and within the previously defined OUT-zone (red). Grey event are located outside both regions, and yellow dots correspond to the seismicity with M < 4. Both grey and yellow events are not taken into account for the seismic rate calculations. (**c–e**) Variations as a function of time of the cumulative number of events for both areas (IN-zone, blue curves) and the surrounding zone (OUT-zone, red curves). Only the events with M > 4 are considered. Dashed lines correspond to the observed seismic rates. (**c**) 2000–2010 pre-mainshock period. (**d**) Full 2000–2019 period. (**e**) Histograms of the seismic density in both IN and OUT zones.

The 27 February 2010 Maule M_W_ 8.8 earthquake is known to have had very few earthquakes of M_W_ > 5 in the large rupture zone during a long period of time, starting at least in the 1990’s^4,5^. In a preliminary study Madariaga et al*.*^2^ identified only ten events of M_W_ > 5 in the 10-year period before the main shock. During this last part of the interseismic period before the Maule earthquake, the seismic activity (M > 4) within our defined OUT-zone shows a regular trend of about 25 events per year (Fig. 4), followed by a regular trend of about 32 events per year starting a few months after the main-shock, involving a large reactivation zone at depth within the slab. As a result of the Maule GME, the seismic station network substantially improved after 2010 in the studied region, which could explain the increase of ~ 30% of the seismic rate of the OUT-zone. However, as confirmed in Fig. 2, the cumulative FMD of both OUT and entire region (red and black, left panel Fig. 2) shows that the local seismic network was already sufficient to catch the seismicity with M > 4 in this particular zone, confirming a physical process behind the observed rate increase. The IN-zone, on the other hand, shows a different behavior (blue curves, Fig. 4c,d): a seismic rate of around 20 events per year from the years 2000 to 2007, followed by a quiet time of 2–3 years before the main shock, where the seismic rate strongly decreased down to about 7 events per year. Only one event with M_W_ > 5 was recorded during this period, whereas this zone experienced ten M_W_ > 5 in the first 5 years of the twenty-first century, as explained before. Two years after the main shock and its numerous aftershocks, the rupture zone of the Maule earthquake came back to its normal seismic rate value of 20–22 events per year, highlighting the unusual aspect of the seismic quiescence observed between 2007 and 2010. The appearance of a seismic gap during the period from 2007 to 2010 within the IN-zone, not observed in the OUT-zone, seems to indicate that the plate-interface locally locked, entailing an increase of the stress acting on the major patches of “silent” locked asperities, located within the IN-zone. Indeed, deeper earthquakes are still observed during this period (east part of the OUT-zone, Figs. 1b and 4a), indicating that processes of slab plunge continues, while the upper part is resisting the traction. Furthermore, various geodetic studies confirm that before the main shock, the locking degree was indeed very high at the coseismic interface^36,42,43^. The quiescence of the IN-zone being not “absolute”, slip at the interface probably still occurs, releasing a small part of the accumulated energy.

### Foreshock activity

**Figure 5:**
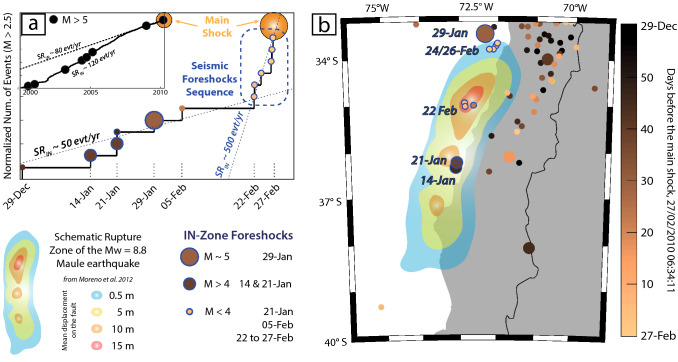
Early change of the seismic rate before the M_W_ 8.8 Maule main shock. (**a**) Normalized number of events within the rupture zone (IN-zone). The upper left-corner plot shows the long time-scale seismic activity as a function of time. (**b**) Distribution of the recorded events in the last 2 months before the main shock. Red and orange areas correspond to the largest co-seismic slip (10–16 m, from Moreno et al*.*^36^ finite fault model). The marker face colors indicate the time of occurrence of the events in days before the main shock.

Five to six weeks before the main shock, two events with M > 4 where recorded within the rupture zone on 14 and 21 January, as already observed by Bouchon et al*.*^3^, followed a week later by a M_W_ = 5 event very close to the previously defined IN-zone, on 29 January 2010 (Fig. 5a). Both M > 4 events were located very close to the epicenter of the 2010 earthquake in front of the town of Cobquecura. Slip in this patch was close to 10 m during the main shock (Fig. 5b). After January 29 (day of the last M_W_ = 5 foreshock), a decrease of about a month in the activity within the IN-zone was observed, until the initiation of a seismic reactivation displayed by two foreshock bursts on 22 and 24–26 February. The first one was located near Constitución within the patch that experienced the maximum coseismic displacement in 2010, reaching more than 15 m of slip^36^. This zone remained totally quiet during 10 years before 22 February, except for a M_W_ > 5 event that occurred in 2002, probably showing the existence of a ready-to-slip patch. The second foreshock burst was located at the northern part of the IN-zone, corresponding to the most active nest of seismicity of the rupture area which, after a semi-quiescence time of a few years, experienced the unique M > 5 foreshock of this zone on 29 January 2010, then followed by a quick reactivation a few days before the Maule event (Fig. 5b). These events can be considered as foreshocks of the Maule main event, as the probability of such seismic rate changes in this zone is extremely low (*p* < 0.1% for the IN-zone, and *p* < 0.01% if we only focus on the major coseismic displacement patch). After the main shock, this zone remained the most active, as observed in Fig. 4b (and Supplementary Fig. S1).

### Postseismic sequence

Immediately after the main shock, the seismic rate within the IN-zone drastically increased, concentrating most of the aftershocks sequence^7,8^, and confirming the location of the major seismic coalescent patches at the interface. Two to three years after the main shock, the seismic rate within the IN-zone returned to its initial interseismic value, whereas the OUT-zone presented a slight increase probably connected with the post-seismic reactivation of preexisting seismic faults (Fig. 4 and Supplementary Fig. S1). Interestingly, during the period 2013–2018, while the OUT-zone presents an increase of its seismic rate for M > 4, a decrease of its seismic rate for M < 4 can be observed (Fig. 1b, three upper panels versus three lower panels, and yellow dots Fig. 4a,b). As the seismic network improved during this period compared to the 2000–2009 time window, this decrease of the small earthquake rate in this region is unlikely to be connected to a lack of data. Deeper large earthquakes (z > 50 km) are indeed known to have anomalously low aftershock productivity, specifically in the Andean subduction region^44,45^ due to the temperature and stress regime a depth, leading to a deficit of lower earthquakes magnitudes, compared to medium-to-high magnitudes.

## Discussion

**Figure 6:**
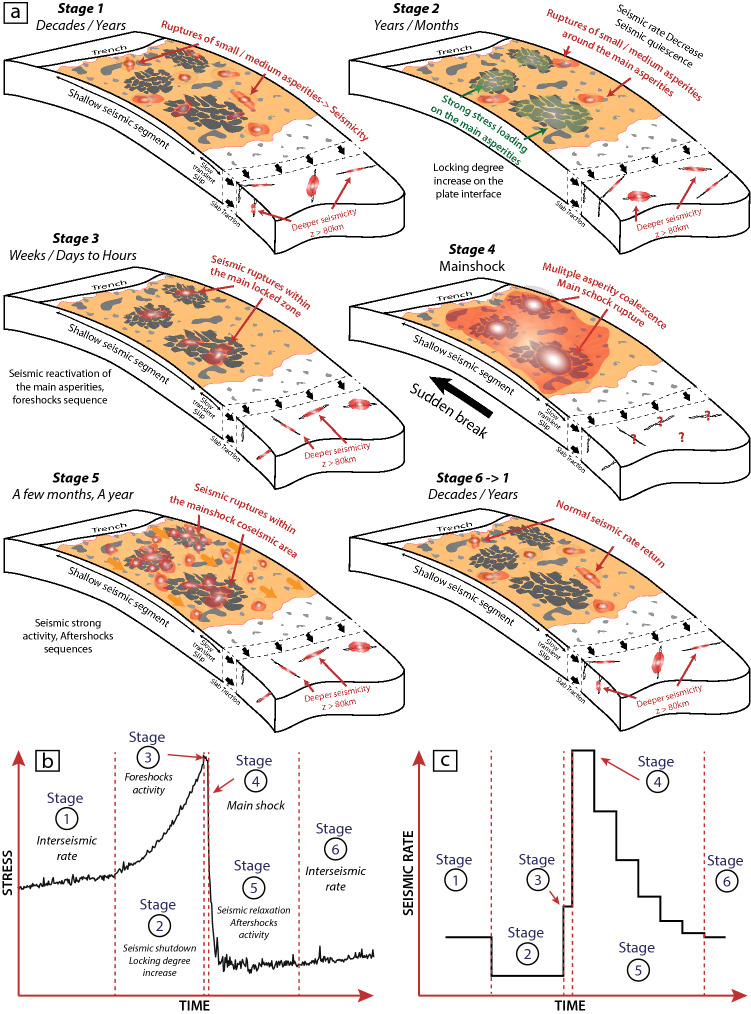
General scheme of the seismic processes before the Maule main shock. (**a**) Stage 1 corresponds to the normal seismic rate connected to the continuous slab traction on the locked zone (…–2007). On Stage 2 the seismic rate decreases, and very few events with M > 5 are observed within the future rupture zone (2007–2010). The locking degree increases at the interface, and the stress strongly increases on the main asperities. Stage 3 corresponds to the foreshock phase occurring a few days before the main shock. This activity occurred close to the areas where the maximum co-seismic slip were observed, indicating the maturity of the main asperities to fail soon, and Stage 4 images the main shock occurring on 27 February 2010. On Stage 5, numerous aftershocks occur during the early relaxation phase within the main shock rupture zone, before the main asperities grip again, increasing the local locking degree. Stage 6. At this stage, the seismic rate reaches its steady-state, going back to the first stage. (**b**) Conceptual scheme of the stress change on the plate interface during the seismic cycle of the M_W_ 8.8 Maule earthquake. (**c**) Conceptual scheme of the seismic rate change.

The observation of pre-seismic quiescence within the 2010 Maule earthquake rupture zone during 2 years, followed by a seismic reactivation a few weeks and days before the main shock, confirm that this megathrust earthquake was preceded by preparation phases reflecting various states of deformation accommodation, and leading to the major rupture it experienced on 27 February 2010 (Fig. 6). The seismic quiescence, illustrated by the stage 2 in Fig. 6, could be interpreted as a period of SSE or ETS^25,31^, but no geodetic data support the existence of such patterns in this zone. Rather, several studies have shown that the coseismic 27F zone was strongly locked before the main shock^6,36,43^, experiencing a very low strain rate compared to the surrounding zones^46^. During the period of low seismic rate, the largest asperities on the plate interface then resist to slip, increasing the locking degree of the entire interface. Such pattern of stress and strain rate changes along the plate interface years before a main shock is in good accordance with some previous studies in other contexts of subduction zones^47,48^.

A short time before the rupture, bursts of seismicity occur within the main locked zones corresponding to the largest ready-to-slip friction patches, and reflecting the high degree of accumulated stress in these places. These foreshocks, that occur in localized areas that are usually quiet, are unlikely to trigger the strong instability resulting in a large rupture, because of the very small Coulomb stress changes involved (only a few mbars of stress change in the case of M < 4), but seem to indicate the state of maturity of the contact. After the main rupture, the rapid deceleration of the seismic rate, reaching its regular rhythm in less than 3 year inside the coseismic area, reflects that the plate interface rapidly re-locked^49,50^ in its quasi-entire surface.

The irregularity of the time scale with which these different preparation phases occur during the seismic cycle has been observed for past large earthquakes^31,51,52^, as well as foreshock occurrence^3,53,54^. However, the sequence of seismicity-quiescence-foreshocks-mainshock-aftershocks-seismicity scheme, illustrated by substantial seismic rate changes (Fig. 6c), similar to the “A-type Mogi Doughnut” seismic pattern observed for medium-to-large earthquakes^51,54^, has never been documented at the scale of a subduction GME. In the case of the M_W_ 8.8 Maule earthquake, these first observations of changes in the seismic rate at different time and space scales, put in evidence variations in the strain accommodation associated to stress concentrations in locked regions of the plate interface, that result in a giant subduction earthquake. As for this earthquake, real time observation of a seismic reactivation in usually quiet zones embedded within a large locked area, associated with unusually rapid seismic reactivation, could represent a last warning before a larger destructive rupture. To monitor a largely locked zone and compare the seismicity of the seismogenic plate interface with the behavior of the surrounding area could therefore be of interest to mitigate seismic risk in the future.

## Supplementary Information

Supplementary Information.

## Data Availability

All the data used in this study come from the seismic catalog made and updated by the International Seismological Center (ISC), and are available at http://www.isc.ac.uk.
