# Supplementary material for: Seismic rate variations prior to the 2010 Maule, Chile MW 8.8 giant megathrust earthquake
Source: Sci Rep. 2021 Feb 1;11:2705. doi: 10.1038/s41598-021-82152-0 (PMC7851146; doi:10.1038/s41598-021-82152-0)
Supplement: Supplementary file 1 — Supplementary Information. [file 41598_2021_82152_MOESM1_ESM.docx]

**SUPPLEMENTARY INFORMATION**

**Seismic rate variations prior to the 2010 Maule, Chile M_W_ 8.8 giant megathrust earthquake**

**Benoit Derode ^1*^, Raúl Madariaga ^1,2^, and Jaime Campos ^1^**

*^1^ Department of Geophysics (DGF), University of Chile, Blanco Encalada 2002, Santiago, Chile*

*^2^ Laboratoire de Géologie, PSL University, Ecole Normale Supérieure et CNRS, 75230 Paris, France*

Corresponding Author: [benoit.derode@gmail.com](mailto:benoit.derode@gmail.com) (Benoit Derode)

**Supplementary Table T1.** Calculated *M_C_* and *b*-value for each period and region defined in Figure 1 (main manuscript) with both Maximum curvature (MAXC) and b-value stability (MBS) methods.

|  |  | **Before**  **(2000-2009)** | | **27F Coseismic**  **2010** | | **After**  **(2011-2018)** | |
| --- | --- | --- | --- | --- | --- | --- | --- |
|  |  | **Mc** | **b** | **Mc** | **b** | **Mc** | **b** |
| **IN** | *MAXC (non-param. method)* | 3,5 | 1,34 | 3,7 | 1,1 | 3,1 | 1 |
|  | *MBS (param. method)* | 3,6 | 1,44 | 3,7 | 1,03 | 3,2 | 1 |
| **OUT** | *MAXC* | 3,4 | 1,16 | 3,5 | 1,12 | 2,9 | 1,04 |
|  | *MBS* | 3,3 | 1,14 | 3,7 | 1,1 | 3,8 | 1,22 |
| **All** | *MAXC* | 3,4 | 1,18 | 3,7 | 1,05 | 3,1 | 1 |
|  | *MBS* | 3,6 | 1,25 | 3,7 | 1 | 3,2 | 1 |


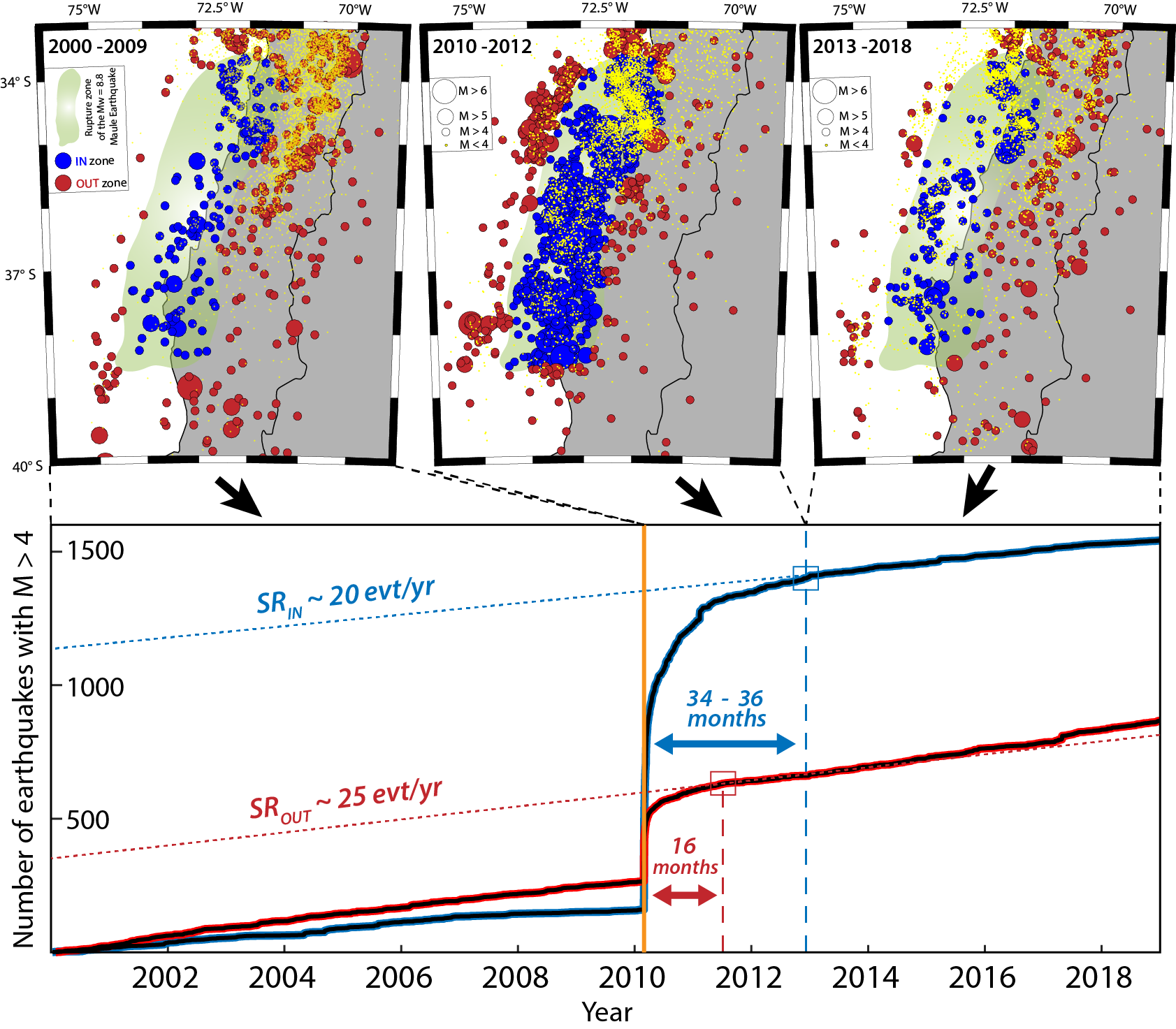


**Supplementary Figure 1. Total seismicity recorded from 2000.** Upper panel, from left to right: Seismicity recorded before (Fig. 4a, main manuscript), during, and after (Fig. 4b, main manuscript) the main Maule event. Lower panel: Cumulative number of events with Mw > 4. The seismic rate of the IN-zone (blue dotted line) returns to its interseismic value after a period of around three years, whereas the OUT-zone seismic rate (red dotted line) decelerates more rapidly, going back to its interseismic value in around 16 months.
